# Partial loss of the vitellogenins confers a fitness disadvantage but does not impact brood size in *C. elegans*

**DOI:** 10.17912/micropub.biology.001789

**Published:** 2025-09-10

**Authors:** Monica M. Macharios, Yasmine D. Hernandez, Peter C. Breen, Robert H. Dowen

**Affiliations:** 1 Integrative Program for Biological and Genome Sciences, University of North Carolina at Chapel Hill, Chapel Hill, North Carolina, United States; 2 Department of Biology, University of North Carolina at Chapel Hill, Chapel Hill, North Carolina, United States; 3 Department of Cell Biology and Physiology, University of North Carolina at Chapel Hill, Chapel Hill, North Carolina, United States

## Abstract

Organismal homeostasis relies on balancing cellular metabolic decisions with environmental conditions, especially during reproduction. Using *Caenorhabditis elegans,* we tested whether vitellogenesis, or the deposition of lipid-rich yolk into oocytes, is required for reproductive output and metabolic balance by creating a strain lacking five of the six vitellogenin genes ( *vit-1/3/4/5/6* ). This mutant produced embryos with reduced lipid content compared to wild-type, but the total brood size remained unaffected, unlike the *rme-2* mutant, which lacks the yolk receptor. However, progeny survival during L1 starvation was impaired in *vit-1/3/4/5/6* animals. This strain offers a new model for studying how vitellogenesis impacts reproductive and organismal fitness.

**Figure 1. Creation and characterization of a strain carrying mutations in five of the six vitellogenin genes:** ( **A** ) A schematic illustrating the nonsense mutations introduced into the *vit-3* , *vit-4* , *vit-5* , and *vit-6* genes by CRISPR/Cas9 editing. The edits to *vit-3* , *vit-4* , and *vit-5* were performed simultaneously using a single crRNA that targeted the same sequence in all three genes. The *vit-3-6* mutations were introduced into the *vit-1(ok2616)* mutant to generate the *vit-1/3/4/5/6* quintuple mutant. ( **B** ) An SDS-PAGE gel containing protein lysates from wild-type, *rme-2(b1008)* , *vit-2(ok2616) vit-1(ok3211)* , and *vit-1/3/4/5/6* animals (25 individuals per lane) stained with Coomassie blue R-250. The arrows indicate bands corresponding to the major yolk proteins (YP170, YP115, and YP88). ( **C** ) Quantification of Nile Red fluorescence in embryos from three independent experiments (mean ± SD; *n* =50/genotype; ns, not significant, *, *P* <0.05, **, *P* <0.01, ***, *P* <0.001, ****, *P* <0.0001, one-way ANOVA). ( **D** ) Brood size measurements for wild-type, *rme-2(b1008)* , *vit-2(ok2616) vit-1(ok3211)* , and *vit-1/3/4/5/6* animals. Eleven individual broods were counted for each genotype (mean ± SD; ns, not significant, **, *P* <0.01, ****, *P* <0.0001, one-way ANOVA). ( **E** ) A time course of survival during L1 starvation (3 independent trials, mean ± SEM reported; *P* values were calculated using a Mantel-Cox log-rank test).

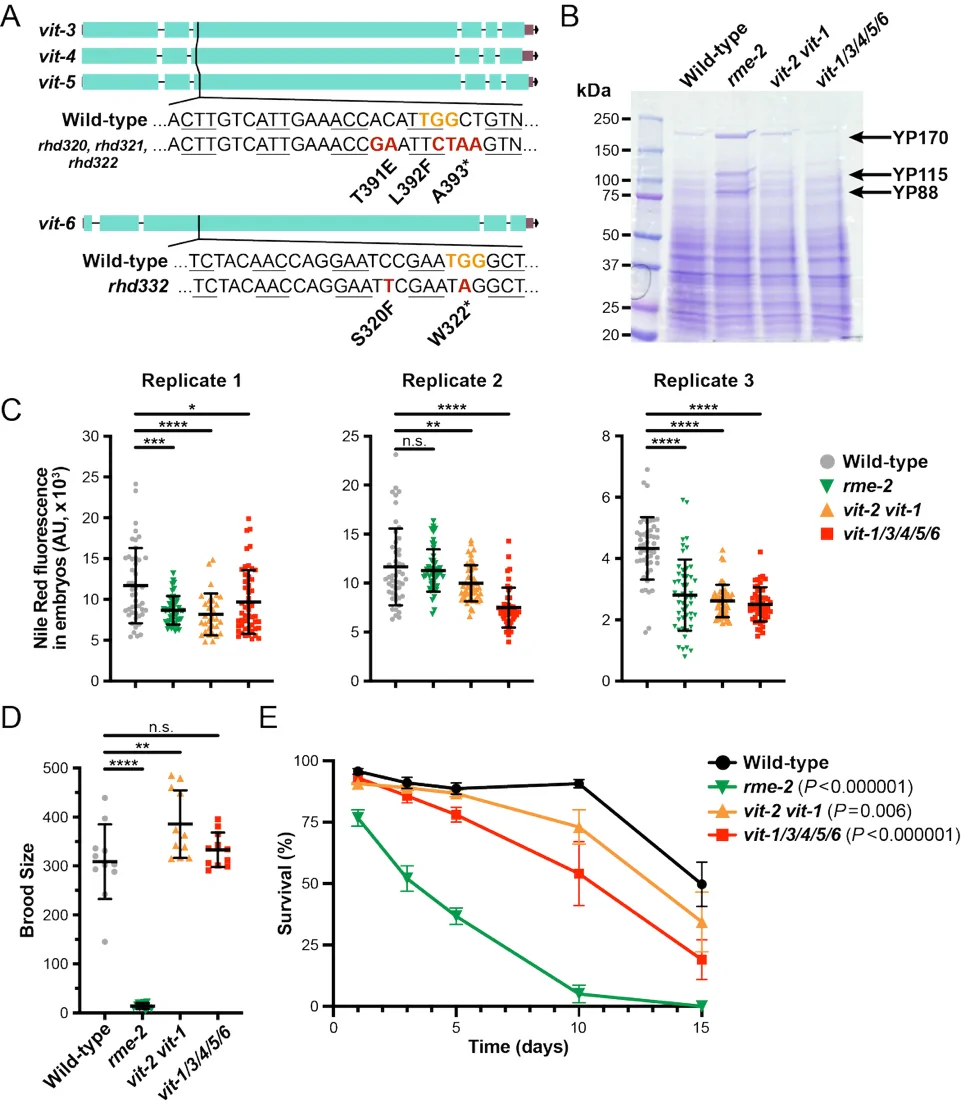



## Description

Vitellogenesis is the process by which nutrients are provisioned to an animal's progeny in the form of yolk, comprised of lipids and vitellogenin lipoproteins (Kimble and Sharrock 1983; Klass et al., 1979; Sharrock 1983; Sharrock et al., 1990). The nematode *C. elegans* possesses six vitellogenin-encoding ( *vit* ) genes, which produce proteins YP170B, encoded by *vit-2 ,* YP170A, encoded by *vit-3 , vit-4 ,* and *vit-5* , and YP115 and YP88 formed from the cleaved *vit-6* gene product (Sharrock 1983; Sharrock et al., 1990). The *vit-1* gene is 82% identical to *vit-2* (Blumenthal et al., 1984; Perez and Lehner 2019); however, it remains unclear if the mature VIT-1 protein is also 170 kDa. The VIT proteins recruit and transport phospholipids, free fatty acids, and cholesterol in the form of lipoprotein particles from the animal's intestine to the mature oocytes in the gonad (Grant and Hirsh 1999; Hall et al., 1999; Kimble and Sharrock 1983; Perez and Lehner 2019; Sharrock et al., 1990). Lipoproteins are internalized by oocytes via the RME-2 receptor through receptor-mediated endocytosis (Grant and Hirsh 1999). Together, the vitellogenin proteins, which are functional orthologues of the human low-density lipoprotein ApoB (Baker 1988), are required for proper delivery of yolk to the progeny. To date, the *vit* genes have only been studied individually or tested in tandem using double loss-of-function mutants or RNAi (Ezcurra et al., 2018; Geens et al., 2023; Murphy et al., 2003; Seah et al., 2016; Sornda et al., 2019).

To further understand the physiological consequence of partial loss of vitellogenin protein, we generated a novel strain that contains loss-of-function mutations in five of the six vitellogenin genes ( *vit-1/3/4/5/6* ). Taking advantage of the high sequence similarity of the *vit-3* , *vit-4* , and *vit-5* genes, we simultaneously engineered a premature stop codon into these three *vit* genes using CRISPR/Cas9 genome editing ( Figure 1A ). These nonsense mutations were introduced into the *vit-1(ok2616)* mutant, yielding the *vit-1/3/4/5* mutant. After verifying the quadruple mutant strain by genotyping and Sanger sequencing of each locus, we engineered a premature stop codon mutation into the *vit-6* locus via CRISPR/Cas9 ( Figure 1A ), thereby generating the *vit-1/3/4/5/6* quintuple mutant.

To validate that our loss-of-function alleles reduced VIT protein production, we stained SDS-PAGE gels containing protein lysates from wild-type animals, the *rme-2(b1008)* mutant, the *vit-2 vit-1* double mutant, and the *vit-1/3/4/5/6* quintuple mutant with Coomassie blue to visualize the highly abundant yolk proteins ( Figure 1B ). Yolk proteins have established molecular weights of 170 kDa (YP170A and YP170B), 115 kDa (YP115), and 88 kDa (YP88) and are easily visualized by Coomassie staining of SDS-PAGE gels (Sharrock 1983). The *rme-2 ( b1008 )* mutant displayed increased VIT protein levels compared to wild-type, which is expected given that oocytes lacking the RME-2 receptor are unable to clear yolk from the body cavity (Grant and Hirsh 1999). In contrast, the *vit-1/3/4/5/6* quintuple mutant displayed severe reductions in VIT protein levels compared to wild-type ( Figure 1B ). These results are consistent with previous studies, where animals subjected to *vit-5* and/or *vit-6* RNAi showed a decrease in the amount of yolk proteins relative to controls when analyzed by Coomassie blue staining (Geens et al., 2023; Sornda et al., 2019). Notably, the *vit-2 vit-1* double mutant showed little impairment in yolk protein synthesis, suggesting that *vit-3-6* may compensate for their loss. Furthermore, we did not observe any increase in YP115 or YP88 levels in the *vit-2 vit-1* double mutant, which has been previously observed in older adult animals subjected to *vit-5* RNAi (Sornda et al., 2019). While we are confident that the *vit-1/3/4/5/6* mutations severely decrease yolk production, we predict that synthesis of YP170B, encoded by the *vit-2* gene, remains unaffected in this strain. Moreover, we are unable to eliminate the possibility that some VIT-3/4/5/6 protein is synthesized in these animals due to stop codon readthrough.

Given that yolk is responsible for the deposition of lipids into the mature oocyte, we hypothesized that partial loss of the *vit* genes would result in lower amounts of embryonic lipids. Using Nile Red to stain neutral lipids and triglycerides, we found that the *vit* mutant embryos exhibited lower amounts of Nile Red fluorescence relative to wild-type across three independent experiments, indicating that lipid levels were reduced in *vit* mutant embryos ( Figure 1C ). We observed a similar phenotype in the *rme-2* mutant. Notably, we only found an average reduction of 32% and 21% in Nile Red staining for the *vit-1/3/4/5/6* and the *rme-2* mutant, respectively, suggesting that additional mechanisms of lipid deposition or *de novo* synthesis compensate for loss of vitellogenesis.

Despite its role in intergenerational nutrient allocation, it has been previously reported that reduced vitellogenin gene expression does not dramatically alter progeny production (Dowen 2019; Ezcurra et al., 2018; Van Rompay et al., 2015). However, animals lacking *rme-2* have dramatically reduced brood sizes (Dowen 2019; Grant and Hirsh 1999). Therefore, we measured the brood size of the *vit-2 vit-1* double mutant and the *vit-1/3/4/5/6* quintuple mutant and compared them to the broods produced by wild-type and *rme-2* animals ( Figure 1D ). Intriguingly, the *vit* mutants produced near wild-type broods while the *rme-2* mutant was nearly sterile, suggesting that the brood size defect of *rme-2* animals is not solely explained by the lack of yolk protein delivery. These results are consistent with those previously described for other vitellogenesis mutants, such as *vrp-1* and *ceh-60* (Dowen 2019; Van Rompay et al., 2015). Finally, our data suggest that RME-2 plays multiple roles in the germline, consistent with previous findings that it also functions in spermathecal valve dilation and ovulation (Chi and Reinke 2009; Grant and Hirsh 1999).

Some vitellogenesis mutants have been shown to have deficiencies in maintaining progeny fitness, including a reduced ability to survive starvation at the L1 larval stage (Chotard et al., 2010; Geens et al., 2023; Van Rompay et al., 2015). Consistently, wild-type larvae that receive a lower dose of yolk as embryos have reduced fitness relative to their siblings that receive higher doses (Perez et al., 2017). Thus, we tested whether our *vit* mutants displayed reduced survival during L1 starvation. Indeed, the *vit-1/3/4/5/6* mutant exhibited lower L1 survival (54%) compared to wild-type (91%) at day 10 of starvation, which persisted to day 15 ( Figure 1E ). The *vit-1/3/4/5/6* survival phenotype was more severe than that of the *vit-2* *vit-1* double mutant; however, no mutant reached the levels of *rme-2* mutant, which had a mean survival of 5% by day 10 and no surviving progeny by day 15 ( Figure 1E ). Together, these data indicate that reduced yolk provisioning, conferred by either mutation of the *vit-1/3/4/5/6* genes or *rme-2* , impairs L1 starvation survival.

It is surprising that the *vit-1/3/4/5/6* mutant does not closely phenocopy the *rme-2* mutant, as both mutants display a deficit in yolk provisioning to the offspring. The dramatic difference in brood size can perhaps be accredited to a defect in the spermatheca valve leading to oocyte damage in the *rme-2* mutant (Chi and Reinke 2009); however, an explanation for why the *rme-2* mutant displays more severe L1 survival phenotypes compared to the *vit-1/3/4/5/6* mutant requires additional studies. One intriguing possibility is that the RME-2 receptor facilitates the uptake of molecules other than yolk. A recent study found that 5-carboxyfluorescein (5-CF), a small membrane-impermeable fluorescent molecule, can be transported from the intestine to the oocyte via RME-2 (Turmel-Couture et al., 2024). While it is possible that 5-CF is transported to the germline within yolk particles, it is also possible that RME-2 mediates the uptake of molecules independent of yolk. Thus, additional research is needed to elucidate the full suite of molecules that can be endocytosed by RME-2 in the oocytes.

The *vit-1/3/4/5/6* mutant strain provides a useful avenue to explore the metabolic dysfunction in vitellogenin-depleted animals. Using Nile Red staining, we found that upon reduction of vitellogenin protein, embryos still have a substantial amount of lipids, suggesting that multiple lipid synthesis pathways contribute to maternal nutrient provisioning. It is possible that VIT-2 alone is sufficient to deliver lipids to oocytes in the *vit-1/3/4/5/6* mutant via the established vitellogenesis pathway. However, an alternative mechanism for maternal deposition of lipids into the *C. elegans* germline is through the delivery of malonyl-CoA via gap junctions. Malonyl-CoA is produced in the somatic sheath cells and transported out of the somatic cells by the INX-8 / INX-9 hemichannels and into the germline by the INX-14 / INX-21 hemichannels (Starich et al., 2020). Malonyl-CoA can then be used in the germline and embryos to fuel fatty acid synthesis via *de novo* lipogenesis, which is critical for proper embryonic development (Starich et al., 2020). Perhaps this pathway is heavily utilized when yolk protein expression is reduced, as in the *vit-1/3/4/5/6* or *rme-2* mutants.

Here, we used CRISPR-Cas9 genome editing to generate a novel mutant strain containing nonsense mutations in the *vit-3* , *vit-4* , *vit-5* , and *vit-6* genes, which were combined with the previously established *vit-1(ok2616)* loss-of-function mutation, to yield the quintuple *vit-1/3/4/5/6* mutant. Our results indicate that depletion of five of the six vitellogenin proteins does not confer an abnormal brood size, but rather, the progeny exhibit a reduction in fitness during L1 starvation. While our well-fed conditions support efficient propagation of this strain in the laboratory, it is likely that these animals would be at a severe disadvantage in the wild where maternal provisioning of lipids is likely crucial for progeny survival during adverse or stressful conditions. This strain will be a useful resource to other research labs interested in investigating the metabolic tradeoffs during development, reproduction, and aging.

## Methods

*Maintenance and generation of C. elegans strains*

Animals were reared at 20°C on agar plates containing Nematode Growth Media (Brenner 1974). Plates were seeded with *E. coli* OP50 grown overnight in 2xYT at 37°C. The *vit-2 ( ok3211 ) vit-1 ( ok2616 )* double mutant was created by standard genetic crossing. PCR using locus-specific primers and Sanger sequencing were used to ensure that all mutations were homozygous.

*CRISPR/Cas9 genome editing*

The quintuple mutant vit-6(rhd332[S320F, W322*]) IV; vit-5(rhd322[T391E, L392F, A393*]) vit-4(rhd321[T391E, L392F, A393*]) vit-3(rhd320[T391E, L392F, A393*]) vit-1(ok2616) X was generated using CRISPR/Cas9 genomic editing. First, the T391E/L392F/A393* mutations were simultaneously introduced into the *vit-3* , *vit-4* , and *vit-5* loci using a single crRNA, with the T391E/L392F mutations creating a novel EcoRI restriction site within each locus. The Cas9::crRNA:tracrRNA complexes, as well as the ssODN repair template, were microinjected into the germline of *vit-1(ok2616)* animals to generate DLS976 as previously described (Ghanta and Mello 2020). The *vit-3* , *vit-4* , and *vit-5* edits were independently verified by PCR using locus-specific primer pairs (each with distinct annealing temperatures) followed by an EcoRI digestion, which yielded a unique set of DNA fragments for each locus. The PCR amplicons were also subjected to Sanger sequencing. Next, the *vit-6* locus was edited in DLS976 using the same CRISPR strategy to generate DLS1004 containing the S320F/W322* mutations, which also produce a novel EcoRI restriction site within the *vit-6* locus. Sanger sequencing was performed on all CRISPR mutations to confirm proper editing and to verify homozygosity.

*Coomassie blue staining*

For each genotype, 25 worms (day 2 adults) were harvested into 26 µL of M9 media by picking, snap frozen in liquid nitrogen, and stored at -80ºC. Then, 10 µL of 4X Laemmli sample buffer (Bio-Rad, 1610747) and 4 µL of 1 M Dithiothreitol (DTT) were added to the samples before incubating them at 100°C for 5 minutes. Protein samples were sonicated for 10 minutes using a Bioruptor Pico instrument (Diagenode), incubated at 100°C for an additional 5 minutes, and briefly centrifuged. Sodium dodecyl sulfate–polyacrylamide gel electrophoresis (SDS-PAGE) was performed using 4–20% precast gels (Bio-Rad, 4568094) and Tris/Glycine/SDS running buffer (Bio-Rad, 1610732). Gels were stained with 0.5% Coomassie brilliant blue R-250 (in 45% ethanol, 9% glacial acetic acid, 45% water) with gentle shaking, destained in a 50% methanol, 10% glacial acetic acid, 40% water solution, and finally rinsed in deionized water for at least 1 hour. Gels were imaged using the ImageQuant LAS 4000 instrument (GE Healthcare) and protein bands were identified based on previously published analyses (Sornda et al., 2019). The experiment was performed three times with similar results.

*Nile Red staining*

Nile Red staining was performed on embryos harvested by hypochlorite treatment as previously described (Escorcia et al., 2018). Isopropanol-fixed embryos were stained for 2 hours with a freshly prepared Nile Red/isopropanol solution (60 μL of 0.5 mg/mL Nile Red stock in 940 µL of 40% isopropanol). The embryos were immediately washed, mounted on agar pads, and imaged using a 20X objective on a Nikon Ti2 widefield microscope equipped with a Hamamatsu ORCA-Fusion BT camera. For image quantification, average fluorescence intensities (mean gray values) were measured by manually circling the embryos ( *n* =50 per genotype) using Fiji (version 2.14.0/1.54f). No background subtraction was performed due to a lack of background fluorescence. The data were plotted as the mean ± SD, outlier data were removed using default parameters, and a one-way ANOVA followed by a Bonferroni's multiple comparisons correction was performed using Prism 10. The experiment was independently replicated three times.

*Brood size assay*

Twelve animals per genotype were singled to individual plates each day for five days and allowed to lay embryos. Two days after each transfer, when progeny had grown to the L4 stage, the plate was scored for hatched animals. Any mothers that died prior to the final transfer were censored. The total number of hatched progeny was calculated, the data were plotted as the mean ± SD, and a one-way ANOVA followed by a Bonferroni's multiple comparisons correction was performed using Prism 10.

*Starvation assays*

Hypochlorite treatment was used to isolate embryos from gravid adults and L1s were synchronized in M9 media by overnight rotation at 20°C. The L1 animals were maintained in 15 mL conical tubes containing M9 media without cholesterol (~8 worms/µL) with rotation at 20°C until scoring. On days one, three, five, ten, and fifteen, 100 worms per genotype were dropped onto unseeded plates and scored for movement. Animals that displayed no movement after 10 seconds were considered dead. The experiment was performed three independent times. The percentage of animals alive at each time point was plotted as the mean ± SEM using Prism 10. To compare the survival curves between wild-type and each mutant, we simulated the survival of each genotype for 100 arbitrary individual worms based on the average population-level survival percentages measured at each timepoint and performed a Mantel-Cox log-rank test in Prism, as previously described (Lee and Ashrafi 2008; Zhang et al., 2011).

## Reagents

**PRIMERS**

**Table:** 

**Gene**	**Primer Pair**	**Annealing Temp (ºC)**	**Expected Band Sizes (bps)**
*vit-1(ok2616)*	*For: ATCAGTTGTCCGCGGAATTGRev: GGGACATTGGCTCAACTGTG*	59	WT: 2399MT: 602
*vit-1(ok2616)*	*For: CATCCTCCCAGTCGATACCC Rev: GGGACATTGGCTCAACTGTG*	59	WT: 1445MT: 0
*vit-2(ok3211)*	*For: TCACATGGAAAACGAGGACARev: TGGGATCTTTCCAGAGATGG*	59	WT: 1321MT: 600
*vit-3(rhd320)*	*For: TCCGCTTTTTGCAAAGTATCRev: TGGTTGACGTGGATCTTGGA*	56	- EcoRI: 845+ EcoRI: 386 & 409
*vit-4(rhd321)*	*For: TACTTTCAGGTCTCTGGACCRev: TTTGTCTAGATGCTGGGCGG*	57	- EcoRI: 599+ EcoRI: 342 & 257
*vit-5(rhd322)*	*For: CAGCACACAAGTTTTCAGGTRev: GATGCTCTTCTTCTCGAAGT*	56	- EcoRI: 361+ EcoRI: 351 & 10
*vit-6(rhd332)*	*For: CGCACCCTCGAAGGAGAATGRev: CAAGAGATGGGTAGCGCATG*	59	- EcoRI: 820+ EcoRI: 405 & 415

**CRISPR**

**Table:** 

**Gene(s)**	**crRNA**	**ssODN**
*vit-3, vit-4, vit-5*	ACTTGTCATTGAAACCACAT	GTTCAACTTGTCATTGAAACCGAATTCTAA GTGGCTGGAACCAAGAACACCATTCAACAC
*vit-6*	TCTACAACCAGGAATTCGAA	CCGAGCTTGTCTACAACCAGGAATTCGAAT AGGCTGAGCAACAATGGGCTCAAACTGGAG

STRAINS

**Table:** 

**Strain**	**Genotype**	**Available From**
N2	Wild-type	CGC
DH1390	*rme-2(b1008) IV*	CGC
RB1982	*vit-1(ok2616) X*	CGC
RB2365	*vit-2(ok3211) X*	CGC
DLS882	*vit-2(ok3211) vit-1(ok2616) X*	Upon request
DLS976	vit-5(rhd322[T391E, L392F, A393*]) vit-4(rhd321[T391E, L392F, A393*]) vit-3(rhd320[T391E, L392F, A393*]) vit-1(ok2616) X	Upon request
DLS1004	vit-6(rhd332[S320F, W322*]) IV; vit-5(rhd322[T391E, L392F, A393*]) vit-4(rhd321[T391E, L392F, A393*]) vit-3(rhd320[T391E, L392F, A393*]) vit-1(ok2616) X	Upon request
