# Corrigendum: Partial loss of the vitellogenins confers a fitness disadvantage but does not impact brood size in *C. elegans*

**DOI:** 10.17912/micropub.biology.002461

**Published:** 2026-10-05

## Description

For Macharios MM, Hernandez YD, Breen PC, Dowen RH. 2025. Partial loss of the vitellogenins confers a fitness disadvantage but does not impact brood size in *C. elegans* . microPublication Biology. 10.17912/micropub.biology.001789, the authors have discovered genotyping anomalies for the *vit-2* ( *ok3211* ) allele used in their publication. The discovery impacts the genotype of two strains used in their study. The authors requested corrections to the article to update the genotypes of the strains used, and to rephrase the phenotypes reported in the context of the partial loss of vitellogenin function. The corrections made to the article are as follows:

Title

**Original** : "Loss of the vitellogenins confers a fitness disadvantage but does not impact brood size in *C. elegans"*

**Corrected** : "Partial loss of the vitellogenins confers a fitness disadvantage but does not impact brood size in *C. elegans"*

Abstract

**Original** : "Using *Caenorhabditis elegans* , we tested whether vitellogenesis, or the deposition of lipid-rich yolk into oocytes, is required for reproductive output and metabolic balance by creating a strain lacking all six vitellogenin genes ( *vit-1-6* )."

**Corrected** : "Using *Caenorhabditis elegans* , we tested whether vitellogenesis, or the deposition of lipid-rich yolk into oocytes, is required for reproductive output and metabolic balance by creating a strain lacking five of the six vitellogenin genes ( *vit-1/3/4/5/6* )."

**Original** : "However, progeny survival during L1 starvation was impaired in *vit-1-6* animals."

**Corrected** : "However, progeny survival during L1 starvation was impaired in *vit-1/3/4/5/6* animals."

Figure Title

**Original** : “Creation and characterization of a strain carrying mutations in all six vitellogenin” genes

**Corrected** : “Creation and characterization of a strain carrying mutations in five of the six vitellogenin genes”

Figure Legend

**Original** : “The *vit-3-6* mutations were introduced into the *vit-2(ok3211) vit-1(ok2616)* double mutant to generate the *vit-1-6* sextuple mutant.”

**Corrected** : “The *vit-3-6* mutations were introduced into the *vit-1(ok2616)* mutant to generate the *vit-1/3/4/5/6* quintuple mutant.”

**Original** : “(B) An SDS-PAGE gel containing protein lysates from wild-type, *rme-2(b1008)* , *vit-2(ok3211) vit-1(ok2616)* , and *vit-1-6* animals (25 individuals per lane) stained with Coomassie blue R-250.”

**Corrected** : “(B) An SDS-PAGE gel containing protein lysates from wild-type, *rme-2(b1008)* , *vit-2(ok2616) vit-1(ok3211)* , and *vit-1/3/4/5/6* animals (25 individuals per lane) stained with Coomassie blue R-250.”

**Original** : “(D) Brood size measurements for wild-type, *rme-2(b1008)* , *vit-2(ok3211) vit-1(ok2616)* , and *vit-1-6* animals.”

**Corrected** : “(D) Brood size measurements for wild-type, *rme-2(b1008)* , *vit-2(ok2616) vit-1(ok3211)* , and *vit-1/3/4/5/6* animals.”

Description

**Original** : “To further understand the physiological consequence of total loss of vitellogenin protein, we generated a novel strain that contains loss-of-function mutations in all six vitellogenin genes ( *vit-1-6* ).”

**Corrected** : “To further understand the physiological consequence of partial loss of vitellogenin protein, we generated a novel strain that contains loss-of-function mutations in five of the six vitellogenin genes ( *vit-1/3/4/5/6* ).”

**Original** : “These nonsense mutations were introduced into the *vit-2(ok3211) vit-1(ok2616)* double mutant, yielding the *vit-1-5* mutant.”

**Corrected** : “These nonsense mutations were introduced into the *vit-1(ok2616)* mutant, yielding the *vit-1/3/4/5* mutant.”

**Original** : “After verifying the quintuple mutant strain by genotyping and Sanger sequencing of each locus, we engineered a premature stop codon mutation into the *vit-6* locus via CRISPR/Cas9 (Figure 1A), thereby generating the *vit-1-6* mutant.”

**Corrected** : “After verifying the quadruple mutant strain by genotyping and Sanger sequencing of each locus, we engineered a premature stop codon mutation into the *vit-6* locus via CRISPR/Cas9 (Figure 1A), thereby generating the *vit-1/3/4/5/6* quintuple mutant.

**Original** : “To validate that our loss-of-function alleles impaired VIT protein production, we stained SDS-PAGE gels containing protein lysates from wild-type animals, the *rme-2(b1008)* mutant, the *vit-2 vit-1* double mutant, and the *vit-1-6* sextuple mutant with Coomassie blue to visualize the highly abundant yolk proteins (Figure 1B).”

**Corrected** : “To validate that our loss-of-function alleles reduced VIT protein production, we stained SDS-PAGE gels containing protein lysates from wild-type animals, the *rme-2(b1008)* mutant, the *vit-2 vit-1* double mutant, and the *vit-1/3/4/5/6* quintuple mutant with Coomassie blue to visualize the highly abundant yolk proteins (Figure 1B).”

**Original** : “In contrast, the *vit-1-6* sextuple mutant displayed severe reductions in VIT protein levels compared to wild-type (Figure 1B).”

**Corrected** : “In contrast, the *vit-1/3/4/5/6* quintuple mutant displayed severe reductions in VIT protein levels compared to wild-type (Figure 1B).”

**Original** : “While we are confident that the *vit-1-6* mutations severely decrease yolk production, we are unable to eliminate the possibility that some VIT protein is synthesized in these animals due to stop codon readthrough.”

**Corrected** : “While we are confident that the *vit-1/3/4/5/6* mutations severely decrease yolk production, we predict that synthesis of YP170B, encoded by the *vit-2* gene, remains unaffected in this strain. Moreover, we are unable to eliminate the possibility that some VIT-3/4/5/6 protein is synthesized in these animals due to stop codon readthrough.”

**Original** : “Given that yolk is responsible for the deposition of lipids into the mature oocyte, we hypothesized that loss of the *vit* genes would result in lower amounts of embryonic lipids.”

**Corrected** : “Given that yolk is responsible for the deposition of lipids into the mature oocyte, we hypothesized that partial loss of the *vit* genes would result in lower amounts of embryonic lipids.”

**Original** : “Notably, we only found an average reduction of 32% and 21% in Nile Red staining for the *vit-1-6* and the *rme-2* mutant, respectively, suggesting that additional mechanisms of lipid deposition or *de novo* synthesis compensate for loss of vitellogenesis.”

**Corrected** : ” Notably, we only found an average reduction of 32% and 21% in Nile Red staining for the *vit-1/3/4/5/6* and the *rme-2* mutant, respectively, suggesting that additional mechanisms of lipid deposition or *de novo* synthesis compensate for loss of vitellogenesis.

**Original** : “Therefore, we measured the brood size of the *vit-2 vit-1* double mutant and the *vit-1-6* sextuple mutant and compared them to the broods produced by wild-type and *rme-2* animals (Figure 1D).”

**Corrected** : “Therefore, we measured the brood size of the *vit-2 vit-1* double mutant and the *vit-1/3/4/5/6* quintuple mutant and compared them to the broods produced by wild-type and *rme-2* animals (Figure 1D).”

**Original** : “Finally, our data suggest that RME-2 plays multiple roles in the germline, consistent with previous findings that it also functions in spermathecal valve dilation and ovulation (Chi and Reinke 2009).”

**Corrected** : ”Finally, our data suggest that RME-2 plays multiple roles in the germline, consistent with previous findings that it also functions in spermathecal valve dilation and ovulation (Chi and Reinke 2009; Grant and Hirsh 1999).”

**Original** : “Indeed, the *vit-1-6* mutant exhibited lower L1 survival (54%) compared to wild-type (91%) at day 10 of starvation, which persisted to day 15 (Figure 1E).”

**Corrected** : “Indeed, the *vit-1/3/4/5/6* mutant exhibited lower L1 survival (54%) compared to wild-type (91%) at day 10 of starvation, which persisted to day 15 (Figure 1E).”

**Original** : “The *vit-1-6* survival phenotype was more severe than that of the *vit-2 vit-1* double mutant; however, no mutant reached the levels of *rme-2* mutant, which had a mean survival of 5% by day 10 and no surviving progeny by day 15 (Figure 1E).”

**Corrected** : “The *vit-1/3/4/5/6* survival phenotype was more severe than that of the *vit-2 vit-1* double mutant; however, no mutant reached the levels of *rme-2* mutant, which had a mean survival of 5% by day 10 and no surviving progeny by day 15 (Figure 1E).”

**Original** : “Together, these data indicate that loss of yolk provisioning, conferred by either mutation of the six *vit* genes or *rme-2* , impairs L1 starvation survival.”

**Corrected** : “Together, these data indicate that reduced yolk provisioning, conferred by either mutation of the *vit-1/3/4/5/6* genes or *rme-2* , impairs L1 starvation survival.”

**Original** : “It is surprising that the *vit-1-6* mutant does not closely phenocopy the *rme-2* mutant, as both mutations result in failure to provision yolk to the offspring.”

**Corrected** : “It is surprising that the *vit-1/3/4/5/6* mutant does not closely phenocopy the *rme-2* mutant, as both mutants display a deficit in yolk provisioning to the offspring.”

**Original** : “The dramatic difference in brood size can perhaps be attributed to a defect in the spermatheca valve leading to oocyte damage in the *rme-2* mutant (Chi and Reinke 2009); however, an explanation for why the *rme-2* mutant displays more severe L1 survival phenotypes compared to the *vit-1-6* mutant requires additional studies.”

**Corrected** : “The dramatic difference in brood size can perhaps be accredited to a defect in the spermatheca valve leading to oocyte damage in the *rme-2* mutant (Chi and Reinke 2009); however, an explanation for why the *rme-2* mutant displays more severe L1 survival phenotypes compared to the *vit-1/3/4/5/6* mutant requires additional studies.”

**Original** : “The *vit-1-6* mutant strain provides a useful avenue to explore the metabolic dysfunction in vitellogenin-depleted animals.”

**Corrected** : “The *vit-1/3/4/5/6* mutant strain provides a useful avenue to explore the metabolic dysfunction in vitellogenin-depleted animals.”

**Original** : “Using Nile Red staining, we found that upon loss of the vitellogenin proteins, embryos still have a substantial amount of lipids, suggesting that multiple lipid synthesis pathways contribute to maternal nutrient provisioning.”

**Corrected** : “Using Nile Red staining, we found that upon reduction of vitellogenin protein, embryos still have a substantial amount of lipids, suggesting that multiple lipid synthesis pathways contribute to maternal nutrient provisioning.”

**Original** : “One alternative mechanism for maternal deposition of lipids into the *C. elegans* germline is through the delivery of malonyl-CoA via gap junctions.”

**Corrected** : “It is possible that VIT-2 alone is sufficient to deliver lipids to oocytes in the *vit-1/3/4/5/6* mutant via the established vitellogenesis pathway. However, an alternative mechanism for maternal deposition of lipids into the *C. elegans* germline is through the delivery of malonyl-CoA via gap junctions.”

**Original** : “Perhaps this pathway is heavily utilized when yolk protein expression or function is impaired, as in the *vit-1-6* or *rme-2* mutants.”

**Corrected** : “Perhaps this pathway is heavily utilized when yolk protein expression is reduced, as in the *vit-1/3/4/5/6* or *rme-2* mutants.”

**Original** : “Here, we used CRISPR-Cas9 genome editing to generate a novel mutant strain containing nonsense mutations in the *vit-3* , *vit-4* , *vit-5* , and *vit-6* genes, which were combined with the previously established loss-of-function mutations *vit-1(ok2616)* and *vit-2(ok3211)* , to yield the sextuple *vit-1-6* mutant.”

**Corrected** : “Here, we used CRISPR-Cas9 genome editing to generate a novel mutant strain containing nonsense mutations in the *vit-3* , *vit-4* , *vit-5* , and *vit-6* genes, which were combined with the previously established *vit-1(ok2616)* loss-of-function mutation, to yield the quintuple *vit-1/3/4/5/6* mutant.”

**Original** : “Our results indicate that depletion of all six vitellogenin proteins does not confer an abnormal brood size, but rather, the progeny exhibit a reduction in fitness during L1 starvation.”

**Corrected** : “Our results indicate that depletion of five of the six vitellogenin proteins does not confer an abnormal brood size, but rather, the progeny exhibit a reduction in fitness during L1 starvation.”

Methods

**Original** : “The sextuple mutant vit-6(rhd332[S320F, W322*]) IV; vit-5(rhd322[T391E, L392F, A393*]) vit-4(rhd321[T391E, L392F, A393*]) vit-3(rhd320[T391E, L392F, A393*]) vit-2(ok3211) vit-1(ok2616) X was generated using CRISPR/Cas9 genomic editing.”

**Corrected** : “The quintuple mutant vit-6(rhd332[S320F, W322*]) IV; vit-5(rhd322[T391E, L392F, A393*]) vit-4(rhd321[T391E, L392F, A393*]) vit-3(rhd320[T391E, L392F, A393*]) vit-1(ok2616) X was generated using CRISPR/Cas9 genomic editing.”

**Original** : “The Cas9::crRNA:tracrRNA complexes, as well as the ssODN repair template, were microinjected into the germline of DLS882 to generate DLS976 as previously described (Ghanta and Mello 2020).”

**Corrected** : “The Cas9::crRNA:tracrRNA complexes, as well as the ssODN repair template, were microinjected into the germline of *vit-1(ok2616)* animals to generate DLS976 as previously described (Ghanta and Mello 2020).”

Reagents

**Original** :

Genotyping:

**Table:** 

Alleles	Primer Sequences	Expected Band Sizes (bp)
*vit-1(ok2616)*	*For: AGCGTGAGCTCAAGGAGAAGRev: AGCTTCGTATCCACGACGAC*	WT: 3325MT: 1528
*vit-2(ok3211)*	*For: ATGGAGCACGCTCTTGCTATRev: TGGGATCTTTCCAGAGATGG*	WT: 1378MT: 525
*vit-3(rhd320)*	*For: TCCGCTTTTTGCAAAGTATCRev: TGGTTGACGTGGATCTTGGA*	- EcoRI: 845+ EcoRI: 386 & 409
*vit-4(rhd321)*	*For: TACTTTCAGGTCTCTGGACCRev: TTTGTCTAGATGCTGGGCGG*	- EcoRI: 599+ EcoRI: 342 & 257
*vit-5(rhd322)*	*For: CAGCACACAAGTTTTCAGGTRev: GATGCTCTTCTTCTCGAAGT*	- EcoRI: 361+ EcoRI: 351 & 10
*vit-6(rhd332)*	*For: CGCACCCTCGAAGGAGAATGRev: CAAGAGATGGGTAGCGCATG*	- EcoRI: 820+ EcoRI: 405 & 415

**Corrected** :

Primers

**Table:** 

Gene	Primer Pair	Annealing Temp (ºC)	Expected Band Sizes (bps)
*vit-1(ok2616)*	*For: ATCAGTTGTCCGCGGAATTGRev: GGGACATTGGCTCAACTGTG*	59	WT: 2399MT: 602
*vit-1(ok2616)*	*For: CATCCTCCCAGTCGATACCCRev: GGGACATTGGCTCAACTGTG*	59	WT: 1445MT: 0
*vit-2(ok3211)*	*For: TCACATGGAAAACGAGGACARev: TGGGATCTTTCCAGAGATGG*	59	WT: 1321MT: 600
*vit-3(rhd320)*	*For: TCCGCTTTTTGCAAAGTATCRev: TGGTTGACGTGGATCTTGGA*	56	- EcoRI: 845+ EcoRI: 386 & 409
*vit-4(rhd321)*	*For: TACTTTCAGGTCTCTGGACCRev: TTTGTCTAGATGCTGGGCGG*	57	- EcoRI: 599+ EcoRI: 342 & 257
*vit-5(rhd322)*	*For: CAGCACACAAGTTTTCAGGTRev: GATGCTCTTCTTCTCGAAGT*	56	- EcoRI: 361+ EcoRI: 351 & 10
*vit-6(rhd332)*	*For: CGCACCCTCGAAGGAGAATGRev: CAAGAGATGGGTAGCGCATG*	59	- EcoRI: 820+ EcoRI: 405 & 415

**Original** :

CRISPR Edits

**Table:** 

Gene	crRNA Sequence	ssODN Repair Sequence
*vit-3, vit-4, vit-5*	ACUUGUCAUUGAAACCACAU	GTTCAACTTGTCATTGAAACCGAATTCTAAG TGGCTGGAACCAAGAACACCATTCAACAC
*vit-6*	UCUACAACCAGGAAUCCGAA	CCGAGCTTGTCTACAACCAGGAATTCGAATA GGCTGAGCAACAATGGGCTCAAACTGGAG

**Corrected** :

CRISPR

**Table:** 

Gene(s)	crRNA	ssODN
*vit-3, vit-4, vit-5*	ACTTGTCATTGAAACCGAAT	GTTCAACTTGTCATTGAAACCGAATTCTAA GTGGCTGGAACCAAGAACACCATTCAACAC
*vit-6*	TCTACAACCAGGAATTCGAA	CCGAGCTTGTCTACAACCAGGAATTCGAAT AGGCTGAGCAACAATGGGCTCAAACTGGAG

**Original** :

Strains

**Table:** 

Strain Name	Genotype	Available From
N2	Wild-type	CGC
DH1390	*rme-2(b1008) IV*	CGC
RB1982	*vit-1(ok2616) X*	CGC
RB2365	*vit-2(ok3211) X*	CGC
DLS882	*vit-2(ok3211) vit-1(ok2616) X*	Upon request
DLS976	vit-5(rhd322[T391E, L392F, A393*]) vit-4(rhd321[T391E, L392F, A393*]) vit-3(rhd320[T391E, L392F, A393*]) vit-2(ok3211) vit-1(ok2616) X	Upon request
DLS1004	vit-6(rhd332[S320F, W322*]) IV; vit-5(rhd322[T391E, L392F, A393*]) vit-4(rhd321[T391E, L392F, A393*]) vit-3(rhd320[T391E, L392F, A393*]) vit-2(ok3211) vit-1(ok2616) X	Upon request

**Corrected** :

Strains

**Table:** 

Strain	Genotype	Available From
N2	Wild-type	CGC
DH1390	*rme-2(b1008) IV*	CGC
RB1982	*vit-1(ok2616) X*	CGC
RB2365	*vit-2(ok3211) X*	CGC
DLS882	*vit-2(ok3211) vit-1(ok2616) X*	Upon request
DLS976	vit-5(rhd322[T391E, L392F, A393*]) vit-4(rhd321[T391E, L392F, A393*]) vit-3(rhd320[T391E, L392F, A393*]) vit-1(ok2616) X	Upon request
DLS1004	vit-6(rhd332[S320F, W322*]) IV; vit-5(rhd322[T391E, L392F, A393*]) vit-4(rhd321[T391E, L392F, A393*]) vit-3(rhd320[T391E, L392F, A393*]) vit-1(ok2616) X	Upon request

